# SUMO1 and Defective Spermatozoa Correlate with Endogenous Hydrogen Peroxide and Live Birth Outcome in Intrauterine Insemination Cycles for Unexplained Infertility

**DOI:** 10.3390/ijms241612775

**Published:** 2023-08-14

**Authors:** Ming-Chao Huang, Shu-Ling Tzeng, Wen-Jung Chen, Sung-Lang Chen, You-Ren Ding, Chun-I Lee, Maw-Sheng Lee, Tsung-Hsien Lee

**Affiliations:** 1 Department of Obstetrics and Gynecology, MacKay Memorial Hospital, Hsinchu 30071, Taiwan; had36586@gmail.com; 2 MacKay Junior College of Medicine, Nursing, and Management, Taipei 23741, Taiwan; 3 Institute of Medicine, Chung Shan Medical University, Taichung 40203, Taiwan; cherie@csmu.edu.tw (S.-L.T.); j80179213@gmail.com (Y.-R.D.); msleephd@gmail.com (M.-S.L.); 4 Department of Medical Research, Chung Shan Medical University Hospital, Taichung 40203, Taiwan; 5 Department of Urology, Chung Shan Medical University Hospital, Taichung 40203, Taiwan; mimic1024@gmail.com (W.-J.C.); cshy650@csh.org.tw (S.-L.C.); 6 School of Medicine, Chung Shan Medical University, Taichung 40203, Taiwan; adoctor0402@gmail.com; 7 Department of Obstetrics and Gynecology, Chung Shan Medical University Hospital, Taichung 40203, Taiwan; 8 Division of Infertility Clinic, Lee Women’s Hospital, Taichung 40602, Taiwan

**Keywords:** reactive oxygen species, small ubiquitin-like modifier molecules, unexplained infertility, spermatogenesis, intrauterine insemination

## Abstract

This study aimed to investigate the correlation between hydrogen peroxide (H_2_O_2_), small ubiquitin-like modifier molecules (SUMO), and pregnancy outcomes in couples with unexplained infertility (UI) undergoing intrauterine insemination (IUI) treatment. We prospectively collected semen samples from 56 couples with UI and divided the spermatozoa into motile and immotile fractions by density gradient centrifugation (DSC). Immunofluorescence staining was used to examine the immunostaining and localization of nuclear pore complex (NPC), SUMO1, and SUMO2/3 in spermatozoa. We detected H_2_O_2_ levels by chemiluminescence methods. We found that H_2_O_2_ levels correlated with NPC (neck) (r = 0.400) and NPC (tail) (r = 0.473) in motile sperm fractions. In immotile fractions, H_2_O_2_ positively correlated with NPC (tail) (r = 0.431) and SUMO1 (neck) (r = 0.282). Furthermore, the positive NPC (tail) group had a significantly lower live birth rate than the negative NPC group (17.9% = 5/28 vs. 42.9% = 12/28). In conclusion, H_2_O_2_ positively correlated with SUMO1 (neck) and NPC (tail) in human spermatozoa. The DSC may partially eliminate defective spermatozoa (positive NPC staining); however, if defective spermatozoa remain in the motile fraction, this scenario is associated with a low live birth rate following IUI treatment.

## 1. Introduction

The prevalence of infertility is approximately 8 to 15% globally [1]. Half of the infertility is related to male factors, and 20 to 30% play a sole role [2]. Sperm quality is essential for infertility diagnoses, such as sperm concentration, motility, and morphology [3,4]. However, the etiology of sperm-related infertility is usually unidentified by such basic semen analysis. Recently, the role of reactive oxygen species (ROS) on male or unexplained infertility (UI) has been intensively investigated [5,6,7]. The ROS are highly reactive molecules with an oxygen center. Spermatozoon is one of multiple mitochondria mammal cells to produce sufficient ATP, but high ROS levels may damage cells by the side. Elevated ROS levels may cause damage to sperm DNA, biomembranes, or proteins if intrinsic antioxidants cannot overcome ROS increase [8,9]. Although physiological ROS is a positive trigger for sperm capacitation [10,11] and protein tyrosine phosphorylation [12,13], high ROS-mediated damage to spermatozoa is a major pathology contributing to male infertility [7,14].

For the spermatozoa to acquire its normal function to fertilize after release from the testis, post-translational modification (PTM) is a crucial factor [15,16]. Among all the PTMs within spermatozoa, protein tyrosine phosphorylation is the most widely studied target, followed by small ubiquitin modification (sumoylation) [17]. Sumoylation is a process wherein SUMOs (small ubiquitin modifiers) covalently bind to target proteins by lysine residue with four isoforms: SUMO1, SUMO2, SUMO3, and SUMO4 [18]. SUMO2 and SUMO3 are 95% identical in sequence and are generally called SUMO2/3 [19,20,21]. Sumoylation is thought to play an indispensable role in regulating protein function, including DNA-repair-related proteins [22,23]. In andrology, Vigodner and colleagues suggest that high levels of sumoylation could be a potential marker of defective spermatozoa with abnormal morphology [24]. A previous study even indicates that SUMO1 inversely correlates with sperm progressive motility [25]. It is obvious that the complete maturation of spermatozoa is important for successful fertilization. Among the various causes of male infertility, the production of abnormal or suboptimal spermatozoa is quite common. Therefore, it is important to address the mechanisms at the molecule level of the proteins and post-translational modifications in the spermatozoa.

During sumoylation, pre-SUMOs are cleaved by sentrin-specific peptidase. The C-terminal diglycine on SUMOs is exposed and covalently binds to a cysteine residue on the E1 activating enzyme, then is transferred to ubiquitin-conjugating enzyme 9 (Ubc9) by cysteine residue with E3 SUMO ligase conjugate. In the final step of sumoylation, the SU-MOs/Ubc9/E3 complex is transferred to the target protein for functional alteration [26]. Due to disulfide bond formation between SUMOs and cysteine residue on E1 and Ubc9, sumoylation is susceptible to oxidative stress and is thought to be regulated by ROS [27]. Regulation of sumoylation by ROS is reported as the pathophysiology of some diseases, such as heart failure and brain ischemia [27]. Nevertheless, rare human studies report that ROS produced by spermatozoa could affect sumoylation on immature/mature spermatozoa and be associated with infertility, neither in overt male infertility nor in male partners of couples with UI.

The nuclear pore complex (NPC) is a macromolecular structure that mediates a GTP-facilitated molecular transport mechanism between nuclear and cytoplasmic compartments [28]. It is composed of approximately 30 nucleoporins, and these are structurally organized to form cytoplasmic, inner, and nuclear regions of the NPC [29]. The NPC is believed to play a role in the later stages of sperm maturation, based on the observation of a global redistribution of NPCs to the redundant nuclear envelope compartment in developing spermatids in a mouse model [29,30]. While the condensation of the nucleus progresses, nuclear pores become highly concentrated and pack at the site where the redundant nuclear envelope (RNE) will develop during the maturation phase of the mouse sperm. Nevertheless, the presence of NPC in mature spermatozoa could be a marker of RNE, which is colocalized with SUMOs in spermatozoa [24]. 

Intrauterine insemination (IUI) treatment is initially used for the management of couples with mild male factor infertility or UI, namely, without evident female or male factors. Theoretically, more than 50% of UI may feature suboptimal sperm function. The most common sperm preparation methods for IUI treatment are density gradient centrifugation (DGC) and swim-up [31]. Both methods can separate the motile spermatozoa from the immotile ones. However, the DGC manipulation is associated with an elevation of ROS levels and may exhibit a detrimental effect on sperm quality and function [32]. To the best of our knowledge, there is rare medical literature investigating the relationship among ROS, SUMOs, and pregnancy outcomes in patients with UI. Furthermore, the efficiency of DGC on eliminating of spermatozoa with sumoylation is unknown. 

Because ROS and sumoylation are related to sperm quality and male infertility, we hypothesized that ROS potentially correlates with human sperm sumoylation in UI patients. To clarify the correlation between two potential defective sperm markers—ROS and SUMOs—within spermatozoa, we collected 56 semen samples from male partners of UI couples for semen analysis. In addition, we aimed to determine the correlation between SUMO and sperm function in such couples undergoing IUI treatment. We used NPC as a maker of defective spermatozoa. The presence of SUMO1 and SUMO2/3 were investigated in the spermatozoa from UI couples. After analyzing the sperm quality through basic semen analysis, we separated the sperm cells depending on sperm motility by DGC. We also attempted to elucidate the efficiency of DGC in removing defective spermatozoa (those spermatozoa with elevated levels of NPC or sumoylation).

## 2. Results

### 2.1. Demographic Characteristics and Sperm Quality Parameters

We collected 58 male partners’ semen samples from couples with unexplained infertility and excluded two cases because of incomplete patient information. Sperm parameters for quality assessment include participants’ ages, semen volume, sperm cell concentration, motile sperm ratio, and normal sperm morphology ratio (Table 1). Male ages were 25 to 45, and the median was 34 years old. The volume of semen samples was 2.30 (interquartile range 2.1–4.8) mL. For sperm quality, the sperm concentration was 48.10 (30.18–62.75) million/mL; the percentage of motile sperm in the semen was 82.8 (69.0–94.2)%; and the normal morphology ratio was 6 (4–8)%.

### 2.2. ROS Levels and Immunofluorescence Stain in Sperm Fractions

**Figure 1:**
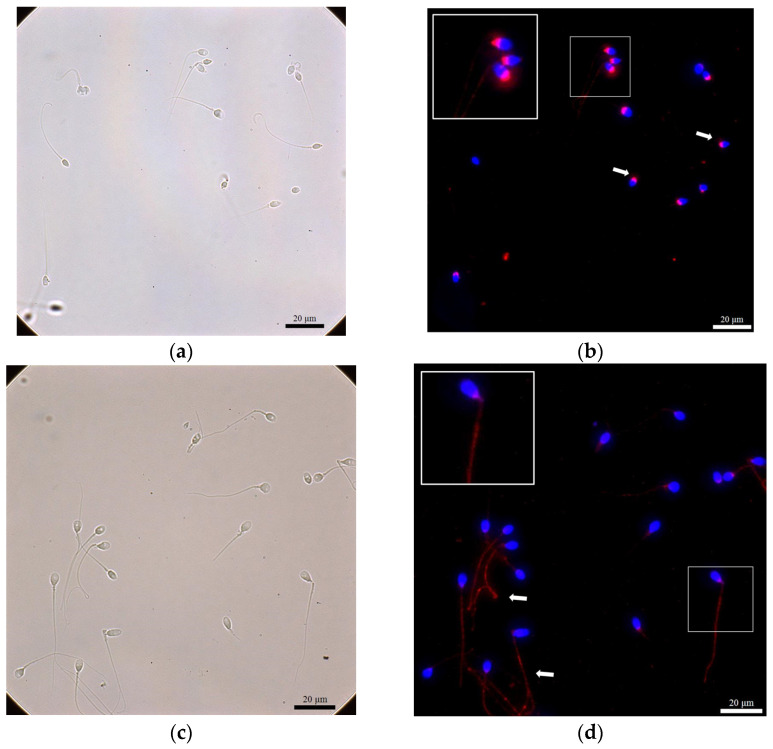
Immunofluorescence stain showed the localization of nuclear pore complex (NPC) at the neck or tail of spermatozoa. (**a**) A bright field for (**b**); (**b**) DAPI (blue) as a nuclear stain, and NPC (red) localized at the neck (arrow). The large white box demonstrated a higher magnification for the area in the small white box. (**c**) A bright field for (**d**); (**d**) DAPI (blue) as a nuclear stain, and NPC (red) localized at the tail of spermatozoa (arrow). The large white box demonstrated a higher magnification for the area in the small white box.

**Figure 2:**
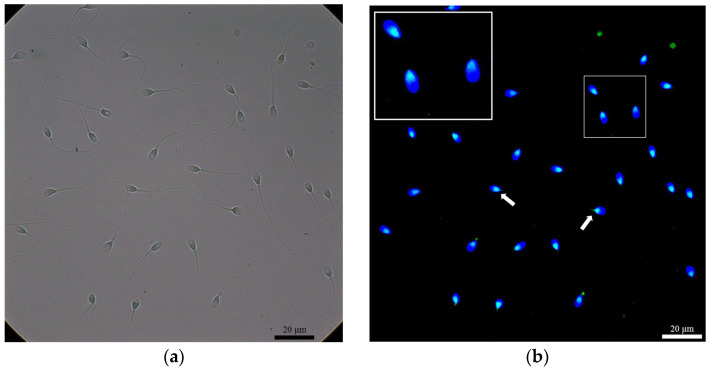
Immunofluorescence stain revealed the immunostaining of SUMO1 at the neck of spermatozoa. (**a**) A bright field for (**b**); (**b**) DAPI (blue) as a nuclear stain, and SUMO1 (green) localized at the neck (arrow). The large white box demonstrated a higher magnification for the area in the small white box.

**Figure 3:**
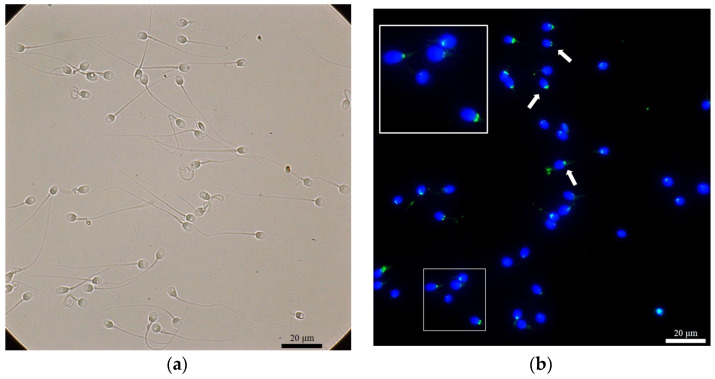
Immunofluorescence stain revealed the immunostaining of SUMO2/3 at the neck (midpiece) of spermatozoa. (**a**) A bright field for (**b**); (**b**) DAPI (blue) as a nuclear stain, and SUMO2/3 (green) localized at the neck (arrow). The large white box demonstrated a higher magnification for the area in the small white box.

We used the density gradient centrifugation (DGC) method to separate motile and immotile sperm fractions, and ROS (H_2_O_2_) levels were detected. In addition, we used the immunofluorescence staining method to detect the presence of NPC, SUMO1, and SUMO2/3 in spermatozoa after DGC preparation (Figure 1, Figure 2 and Figure 3). 

The NPC was revealed at the neck and the tail of spermatozoa (Figure 1). In terms of the NPC (neck) of spermatozoa, 41 samples (73.2%) in the immotile fraction were detected, while 43 samples (76.8%) in the motile fraction were NPC (neck)-positive. We also studied the sumoylation proteins in the nucleus, the neck, and the tail of the spermatozoa in both fractions. SUMO1 was detected only in the neck region (Figure 2), with a detected ratio of 19/56 (33.9%) in the immotile fraction and with a ratio of 13/56 (23.2%) in the motile fraction for the studied patients. When we further investigated the SUMO2/3 (Figure 3) among different locations of sperm in both motile and immotile fractions by positive case numbers, SUMO2/3 (neck)-positive spermatozoa were identified in almost all motile (56/56 = 100%) and immotile (55/56 = 98.2%) fractions.

For the motile and immotile sperm fractions, the H_2_O_2_ levels were 33.23 (26.33–38.42) RLUs and 31.50 (23.97–41.05) RLUs, respectively (Table 2). After DGC, there was no significant difference in the H_2_O_2_ levels between the motile and immotile fractions of the sample. However, the motile fraction spermatozoa, compared with immotile fraction, a higher proportion of NPC, positive spermatozoa were found in the motile fraction compared to the immotile fraction (0.36 (0.20–0.57) vs. 0.13 (0.00–0.27), *p* < 0.001) at the neck, and (0.21 (0–0.42) vs. 0 (0–0.17), *p* < 0.001) at the tail, respectively. However, there was no significant difference in the SUMO1 (neck) levels at both fractions. The proportion of SUMO2/3-positive spermatozoa was also measured, and it showed higher visible SUMO2/3 (neck) in the motile fraction, compared to the immotile fraction (0.73 (0.61–0.84) vs. 0.61 (0.48–0.79), *p* = 0.004) (Table 2).

### 2.3. Correlations between Sperm H_2_O_2_ Levels and Percentages of NPC and SUMO Proteins

To analyze H_2_O_2_ and protein correlation, we used the Spearman correlation test to evaluate the correlation between H_2_O_2_ levels and the percentage of protein (NPC, SUMO1, and SUMO2/3) immunostaining in varied localizations (Table 3). We found that H_2_O_2_ level had a positive correlation with NPC at both neck and tail in the motile sperm fractions (neck: correlation coefficient = 0.400, *p* = 0.002; tail: correlation coefficient = 0.473, *p* < 0.001). SUMO1 in immotile sperm fraction positively correlated with H_2_O_2_ levels (correlation coefficient = 0.282, *p* = 0.035). However, there were no correlations between H_2_O_2_ levels and SUMO2/3 immunostaining in varied localizations. 

To confirm the NPC and SUMOs correlation with H_2_O_2_ levels, we used the Mann–Whitney U test to compare the sperm H_2_O_2_ levels in NPC, SUMO1, and SUMO2/3 at certain subcellular locations (Table 4). We found if NPC could express at the tail, those samples had significantly higher H_2_O_2_ levels in motile (32.85–40.05 vs. 21.51–35.12, *p* < 0.001) and immotile sperm fractions (30.40–49.79 vs. 22.13–36.17, *p* = 0.010), respectively. NPC expressed at the neck had non-significantly higher H_2_O_2_ levels than negative samples in motile and immotile sperm fractions. We also found that cases with SUMO1 (neck) positive have higher H_2_O_2_ levels (32.31–59.97 vs. 23.17–39.40, *p* = 0.031) in immotile sperm fractions. However, we did not find a significant difference in H_2_O_2_ levels for SUMO2/3 (nucleus) positive vs. negative spermatozoa.

### 2.4. The Immunostaining of NPC and SUMO1 Relevant to the Livebirth Outcomes after Intrauterine Insemination for Unexplained Infertile Couples

**Figure 4:**
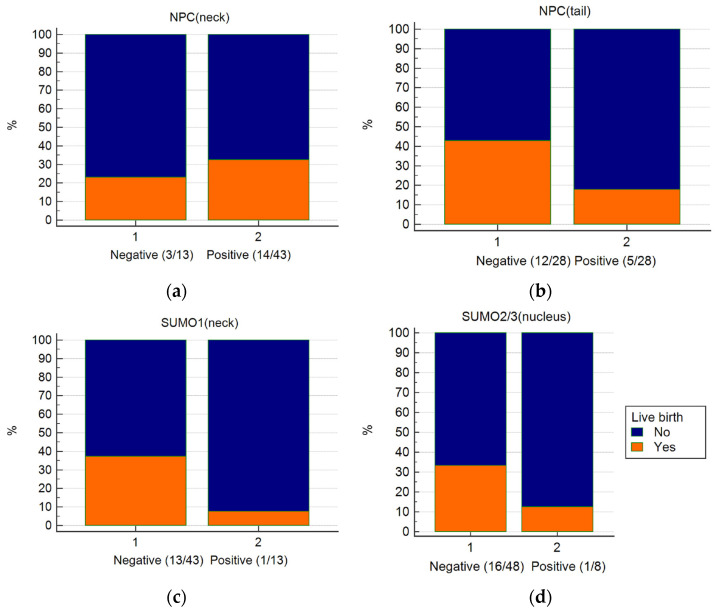
The live birth outcome after intrauterine insemination outcome for couples with unexplained infertility. The couples were divided into groups with or without the presence of NPC and SUMOs in the motile fraction of spermatozoa after density gradient centrifugation. (**a**) NPC (neck); (**b**) NPC (tail); (**c**) SUMO1 (neck); (**d**) SUMO2/3 (nucleus). The results of SUMO2/3 (neck) are not shown because all 56 couples exhibited SUMO2/3 (neck) in the motile fraction of spermatozoa.

At the treatment cycle, the motile fraction after DGC was used for IUI. The motile fraction was divided into positive NPC (tail) spermatozoa (n = 28) and those without NPC (tail) spermatozoa (n = 28) (Figure 4b). The positive NPC (tail) group was also associated with a higher percentage of SUMO1 (neck) spermatozoa (0.00 (0.00–0.50) vs. 0.00 (0.00–0.00), *p* = 0.0059). Furthermore, the positive NPC (tail) group had a significantly lower pregnancy rate compared to that of the negative NPC (tail) group, with 17.9% (5/28) vs. 42.9% (12/28) (*p* = 0.0438, chi-squared test).

We further divided the patients in IUI cycles into positive or negative SUMO1 at the sperm neck in the motile fraction (Figure 4c). Those spermatozoa with positive SUMO1 (neck) were associated with significantly higher NPC (tail) (0.50 (0.25–0.13) vs. 0.00 (0.00–0.50), *p* = 0.0147). Furthermore, the positive SUMO1 (neck) group had a significantly lower pregnancy rate, compared to the negative SUMO1 (neck) group with 7.7% (1/13) vs. 37.2% (16/43) (*p* = 0.0444, chi-squared test).

## 3. Discussion

In this study, we demonstrated that H_2_O_2_ correlated with NPC (tail) and SUMO1 (neck) immunostaining in spermatozoa. The spermatozoa with positive NPC (tail) showed higher H_2_O_2_ levels than the NPC (tail) negative samples, either in the motile or immotile sperm fractions. By contrast, H_2_O_2_ level was positively associated with SUMO1 (neck) only in immotile but not motile sperm fractions. However, we did not find a correlation between H_2_O_2_ levels for SUMO2/3, no matter where it was localized. Interestingly, the SUMO2/3 at the neck of spermatozoa was almost detectable for all the semen samples in both motile and immotile fractions after DGC separation. Furthermore, we found the pregnancy outcome of IUI treatment was correlated with the absence of SUMO1 (neck) and NPC (tail) in motile sperm fractions. 

In physiology, sperm ROS plays an essential role in capacitation to complete fertilization [10,11,33]. Nonetheless, several studies indicate that excessive ROS could damage spermatozoa and have an intimate correlation with infertility in male adults [5,6,7]. From important properties of fluidity and permeability, polyunsaturated fatty acid (PUFA) plays several required roles for flagellar movement and penetration to the oocyte but also makes spermatozoon a target of ROS action [34]. Peroxidation of the plasma membrane by ROS triggers sperm DNA and lipid damage by downstream signal cascade and contributes to mitochondria dysfunction [35]. Then, mitochondrial dysfunction results in the disorganization of sperm axonemes and causes asthenozoospermia [35]. Furthermore, the medical literature also suggests that ROS negatively correlates with sperm concentration and morphology [7,36].

Sumoylation is one of the important PTMs; increasingly more function in mammals’ reproductive system has been identified recently, such as chromatin inactivation [37] and regulation of gametogenesis, including spermatogenesis [38]. In mature human spermatozoa, Marchiani and colleagues revealed that expression of SUMO1 inversely correlates with sperm motility and is mainly located at the nucleus [39]. Nonetheless, Vigodner et al. demonstrated that SUMO1 and SUMO2/3 were highly expressed in the neck, head, and tail regions of spermatozoa using immunofluorescence and electron microscopy [24]. Our results showed varied localization of SUMO1 for the neck dominantly instead of the nucleus. Our result is similar to the results of Vigodner in 2013 [24]. However, our data demonstrated that immotile sperm fractions feature a non-significant higher ratio of SUMO1-positive samples (19/56, 33.9%) than motile sperm fractions (13/56, 23.2%). The results suggest that DGC could not efficiently eliminate the sumoylated spermatozoa, even though the DGC method separates the spermatozoa into motile and immotile fractions depending on varied motility. The efficiency of the swim-up method to eliminate the spermatozoa with sumoylation deserves further investigation.

In the 2010s, Shrivastava and colleagues [40] demonstrated that the primary culture of mouse testis shows an elevated expression of high-molecular-weight (HMW) SUMO1 and SUMO2 conjugate with H_2_O_2_ treatment. In addition, the localization of SUMO1 is also overlapping with double-strand DNA damage caused by H_2_O_2_ in germ cells. Results from previous studies show that high H_2_O_2_ concentrations induce excessive sumoylation [40,41]. Marchiani and colleagues in 2014 demonstrated the correlation between SUMO1 and sperm motility [39]. They suggested that three substrates of SUMO1 in somatic cells, dynamin-related protein, Ran GTPase-activating protein (RanGAP1), and topoisomerase IIα can be sumoylated in human spermatozoa and localize at the midpiece and post acrosome region. They also showed that freezing, thawing, and oxidative stress treatment can increase sumoylation with DNA break increase. Our results also revealed that SUMO1(neck) is positively relevant to H_2_O_2_ levels. Furthermore, the residual SUMO1(neck) spermatozoa within the motile fraction after DGC are detrimental for the pregnancy outcome in IUI cycles for UI couples. 

Even the role of sumoylation, ROS, and the correlation between them has been well studied in mouse models, but less evidence has been reported in infertile couples with normozoospermia. In this study, we describe that human-spermatozoa-endogenous H_2_O_2_ positively correlated with the percentages of SUMO1 immunostaining. Our data also showed that the NPC (tail) of spermatozoa in motile and immotile fractions positively correlated with H_2_O_2_ levels. NPC as a residue envelope marker implies the situation of spermatogenesis when cytoplasm migrates to the sperm neck. Not only does neck localization mean a remaining cytoplasm, but also tail localization indicates the abnormal development of spermatogenesis. We may provide a clue for the regulation of sumoylation and reveal a potential connection between spermatogenesis and the regulation of sumoylation by ROS (H_2_O_2_) in humans with normozoospemia. 

Regarding SUMO2/3, our results showed the neck is the dominant localization, which is in line with the work of Vigodner and colleagues in 2013 [24]. Interestingly, we observed only one human sperm sample without SUMO2/3 immunostaining at the neck region, and the patient featured the highest H_2_O_2_ levels in the present study. Taken together, the SUMO1 may be vulnerable to elevated H_2_O_2_ levels, and the SUMO2/3 may be only affected by extremely high H_2_O_2_ levels. However, we still did not clarify the causality between H_2_O_2_ and SUMO1 in this human study. We may obtain semen samples from male infertility in addition to UI couples to elucidate the connection between ROS, specific H_2_O_2_, and sumoylation in the future.

The NPC and SUMO1 colocalization was demonstrated in previous research with human defective spermatozoa [24]. In the present study, we revealed that the positive NPC (tail) group was also associated with a higher percentage of SUMO1 (neck) spermatozoa. Furthermore, if we used a logistic regression model to adjust the confounding factors, such as female age, BMI, and male age, the presence of NPC (tail) in the motile fractions after DGC was the sole factor correlated with pregnancy outcome in IUI cycles for UI couples. These results indicated that both NPC and SUMO1 were markers for defective spermatozoa. In addition, these markers were relevant to endogenous H_2_O_2_ levels and sperm factor fecundability, even in patients with normozoospermia (UI couples). The clinical significance of NPC and SUMO1 in infertile couples with normozoospermia and other female factor infertility deserves further studies.

The most common sperm preparation methods for IUI treatment are DGC and swim-up. Both methods can separate the motile spermatozoa from the immotile ones based on the motility of the individual spermatozoon [31]. In the swim-up method, we put the semen into the basal layer and covered it with modified HTF media. The motile spermatozoa will swim upward to the media layer. We collected the media layer containing the motile spermatozoa, about 90–120 min later. The present study suggested that the DGC method was ineffective in removing spermatozoa with NPC or SUMO1. Whether the swim-up method could perform better than DGC for IUI treatment requires further investigation.

The limitation of this study is the relatively small sample size. Nonetheless, we recruited the UI couples with strict inclusion criteria. We analyzed in detail the immunostaining of SUMOs and NPC in the motile and immotile fraction of spermatozoa after DGC in this “pure” population with normozoospermia. This is why we could obtain meaningful findings to answer our hypothesis in such a small sample. The second limitation is that we did not use flow cytometry to measure the results of the immunofluorescence stains. However, the subcellular localization of NPC and SUMOs are essential findings in the present study. That is why we used counting methods by human eyes for calculating the percentage of positive stains in the motile and immotile fractions of spermatozoa after DGC.

The strength of this study is that the UI couples, the clinicians, and the technicians for IUI treatment are blind to the results of H_2_O_2_, SUMOs, and NPC in the stage of semen analysis. The live birth outcome of IUI treatments confirmed the clinical value of those findings of NPC and SUMOs in the motile fraction of spermatozoa after DGC for UI couples.

Overall, we revealed the correlation between H_2_O_2_ levels and NPC (tail) and SUMO1 (neck) immunostaining in human spermatozoa from male partners of UI couples. However, we did not find a correlation between H_2_O_2_ and SUMO2/3. Furthermore, the live birth outcome after IUI treatment for UI couples is correlated with the NPC (tail) and SUMO1 (neck) immunostaining in the motile fraction of human spermatozoa after DGC. Such results indicate that H_2_O_2_ levels may regulate the appearance of NPC (tail) and SUMO1 (neck) in human spermatozoa during spermatogenesis and sperm capacitation. Furthermore, the presence of NPC (tail) and SUMO1 (neck) in the motile fraction after DGC correlated with poor sperm performance for UI couples.

## 4. Materials and Methods

### 4.1. Sample Collection, Sperm Quality Analysis, and Sperm Preparation

We collected 58 adult male semen samples at the Reproductive Medicine Center, Chung Shan Medical University Hospital from 17 October 2017 to 27 August 2018 after IRB approval (CS2-17008) in the same hospital. Sperm quality was estimated by a basic semen analysis. Semen samples were collected after 3 to 7 days of abstinence.

We used the Makler Counting Chamber to obtain sperm concentration and motility. The Makler Counting Chamber is a disposable slide with a pre-calibrated grid that allows for accurate sperm counting and motility assessment under a microscope. To assess sperm morphology, we utilized the Papanicolaou staining method, which provides excellent contrast and allows for a detailed evaluation of sperm structure. These techniques ensured the accuracy and reliability of our sperm analysis in the study, widely employed in research and clinical settings to evaluate semen quality and fertility potential.

Semen samples were diluted by PBS depending on sperm concentration from basic semen analysis. Here, to detect ROS (H_2_O_2_ levels) and protein localization from spermatozoa with different motility and preparation for IUI treatment, samples were prepared by the density gradient centrifugation (DGC) method. We prepared a 40/80% gradient solution (PureSperm^®^100; Nidacon, Mölndal, Sweden) and centrifuged it at 3000 rpm for 15 min. Then, we collected interphase (the immotile sperm fraction) and pellets (the motile sperm fraction).

### 4.2. Endogenous H_2_O_2_ Measurement

Endogenous H_2_O_2_ level in samples was detected by chemiluminescence, and luminol (A14597, Alfa Aesar, Haverhill, MA, USA) was used as the probe. We prepared 100 mM luminol as a stocking solution and diluted it into 5 mM as working luminol [42]. The reaction for each well was under 10 μL working luminol and 200 μL sample in a 96-well microplate. Phosphate-buffered saline (PBS) was used as a blank and negative control. The 0.15% and 1.5% H_2_O_2_ were used as positive controls. Luminescent signals were detected by a multi-detection microplate reader system (SpectraMax M5, Molecular Devices, San Jones, CA, USA). Endogenous H_2_O_2_ was detected by kinetic mode for 30 min, and H_2_O_2_ level was defined as the mean of the three highest RLUs for 30 min in this study.

### 4.3. Immunofluorescence Staining of NPC and SUMO Proteins in Spermatozoa

Sperm smears were fixed in 2% to 4% formalin serial 15 min individual. Triton-100X 5% was used for better cell membrane penetration for 30 min. Blocking solutions were added for 30 min for the background mask. Anti-nuclear pore complex (NPC) antibodies (ab24609; 1:200 dilution; Abcam, Cambridge, UK) were used as markers for residual envelope localization and defective spermatogenesis. To detect SUMOs immunostaining in spermatozoa, we used anti-SUMO1 antibody (sc-5308; 1:200 dilution; Santa Cruz, Dallas, TX, USA) and anti-SUMO2/3 antibody (ab3742; 1:200 dilution; Abcam). The primary antibody reaction was under 4 °C overnight. Alexa-488 (1:250; Jackson ImmunoReasearch Inc., West Grove, PA, USA) and 594 (JACKSON 115-585-003; 1:250; Jackson ImmunoReasearch Inc) secondary antibodies were used at room temperature for 1 h. To estimate protein immunostaining level, we calculated at least 200 spermatozoa and recorded the percentage of NPC, SUMO1, and SUMO2/3 immunostaining at subcellular localization. We used a fluorescence microscope (Axio Imager A2, Zeiss, Oberkochen, Germany) to obtain the immunostaining image.

### 4.4. Intrauterine Insemination Treatment

The female partners of couples with unexplained infertility underwent ovulation induction with clomiphene citrate and recombinant FSH (Gona-F, Merck, Darmstadt, Germany). In short, clomiphene (100 mg/day) was used from days 2–4 of the stimulating cycle for five days, followed by gonadotropin (Gona-F, 150 IU/day) treatment until the administration of human chorionic gonadotropin (hCG, Ovidriel, Merck). Transvaginal ultrasound was used to monitor the development of follicles on the 10th–12th day of menstruation. When the leading one or two follicles reached a diameter of 18 mm, hCG (Ovidriel, 250 μg, Merck) was injected for ovulation trigger. Intrauterine insemination treatment (IUI) was performed after 24–36 h. 

On the day of IUI, semen samples were collected after masturbation and processed according to standard procedures specified by the WHO. The same gradient centrifugation method was used to process semen. An IUI tube was used to aspirate the sperm suspension (0.5 mL) from the motile fraction of spermatozoa, the IUI tube was gently inserted into the uterine cavity, and the sperm suspension was slowly injected. After the procedure, the patient was placed in the supine position for 30 min to observe whether there was any special discomfort.

Routine luteal support included oral progesterone (Utrogestan, 400 mg/day) for 14 days, and then all patients took a blood beta-hCG test 14 days after IUI. For those with positive blood HCG (>5 IU), a transvaginal ultrasound examination was performed 7–28 days after the blood test. Clinical pregnancy was confirmed if vaginal ultrasonography detected an intrauterine gestational sac and the fetal heartbeat. Live births were defined as neonates delivered after 24 weeks of gestational age with signs of life within 7 days. 

### 4.5. Statistics

Spearman correlations were used to analyze H_2_O_2_ correlation with sperm quality parameters and protein (NPC, SUMO1, and SUMO2/3) immunostaining score. The Wilcoxon signed rank test was used to compare the difference of percentages of immunostaining-positive spermatozoa between the motile and immotile factions from the same semen (paired samples). The Mann–Whitney U test was used to estimate the significant differences in H_2_O_2_ levels for varied NPC, SUMO1, and SUMO2/3 localization. The X2 test was used to compare of the live birth rates between the spermatozoa with and without the immunostaining of NPC or SUMOs in the motile fraction after DGC. SPSS Statistics 22 software (IBM, Armonk, NY, USA) was used for statistical analysis. A value of *p* < 0.05 was considered significant in all analyses.

## Figures and Tables

**Table 1:** The demographic characteristics of the unexplained infertile couples.

Couples’ Characteristics	Median (25–75%)	Mean ± SD
Female age (years)	32 (30–36)	32.6± 4.7
Male age (years)	34 (32–36.5)	34.8 ± 4.2
Volume (mL)	2.3 (2.1–4.8)	2.5 ± 1.2
Concentration (million/mL)	48.10 (30.18–62.75)	48.98 ± 21.78
Motility (%)	82.8 (69.0–94.2)	79.1 ± 16.8
Morphology (%)	6 (4–8)	6.3 ± 2.4

**Table 2:** Comparison of hydrogen peroxide (H_2_O_2_) levels and sumoylation of spermatozoa in motile and immotile fraction after density gradient centrifugation (DGC) separation. NPC and RLU denote nuclear pore complex and relative light unit, respectively.

N = 56	Motile Fraction		Immotile Fraction		
	Median	25–75%	Median	25–75%	*p* Value ^a^
H_2_O_2_ levels (RLU)	33.23	26.00 to 38.55	31.50	23.82 to 41.63	0.883
NPC at the neck (%)	0.36	0.20 to 0.57	0.13	0.00 to 0.27	<0.001
NPC at the tail (%)	0.21	0.00 to 0.42	0.00	0.00 to 0.17	<0.001
SUMO1 at the neck (%)	0.00	0.00 to 0.00	0.00	0.00 to 0.73	0.174
SUMO2/3 at the nucleus (%)	0.00	0.00 to 0.00	0.00	0.00 to 0.00	0.977
SUMO2/3 at the neck (%)	0.73	0.61 to 0.84	0.61	0.48 to 0.79	0.004

^a^ Wilcoxon signed rank test (paired samples).

**Table 3:** The H_2_O_2_ correlation with the percentages of spermatozoa with NPC, SUMO1, and SUMO2/3 immunostaining. H_2_O_2_ levels and protein correlation were calculated by Spearman correlation tests. The percentage of protein immunostaining was counted by immunofluorescence stain and by counting at least 200 sperm cells.

Protein Localization (%)		H_2_O_2_ Level (RLUs)	
		Motile Fraction	Immotile Fraction
NPC (neck)	Correlation coefficient	0.400	0.172
	*p*-value	0.002	0.205
NPC (tail)	Correlation coefficient	0.473	0.431
	*p*-value	<0.001	0.001
SUMO1 (neck)	Correlation coefficient	0.202	0.282
	*p*-value	0.135	0.035
SUMO2/3 (neck)	Correlation coefficient	0.087	−0.047
	*p*-value	0.523	0.731
SUMO2/3 (nucleus)	Correlation coefficient	−0.247	−0.237
	*p*-value	0.066	0.079

Spearman correlation tests were used for correlation and significance.

**Table 4:** The H_2_O_2_ levels in motile and immotile fractions of DGC-separated spermatozoa with NPC, SUMO1, or SUMO2/3 localization. Positive means we found that protein expressed at certain subcellular locations, and negative means it did not. H_2_O_2_ level was demonstrated by quartile (Q_1_–Q_3_) in the positive or negative group.

Protein Localization			Motile Fraction		Immotile Fraction
		N	H_2_O_2_ Level (RLUs)	N	H_2_O_2_ Level (RLUs)
NPC (neck)	Negative	13	20.17–46.88	15	23.37–75.54
	Positive	43	29.01–38.34	41	26.30–39.64
	*p*-value		0.089		0.560
NPC (tail)	Negative	28	21.51–35.12	34	22.13–36.17
	Positive	28	32.85–40.05	22	30.40–49.79
	*p*-value		<0.001		0.010
SUMO1 (neck)	Negative	43	24.00–38.47	37	23.17–39.40
	Positive	13	33.82–38.36	19	32.11–59.97
	*p*-value		0.070		0.031
SUMO2/3 (neck)	Negative	0	N/A	1	339.15
	Positive	56	26.33–38.42	55	23.90–40.20
	*p*-value		N/A		0.107
SUMO2/3 (nucleus)	Negative	48	27.80–39.68	44	24.27–48.64
	Positive	8	17.99–35.25	12	22.12–32.80
	*p*-value		0.069		0.106

The difference was calculated by the Mann–Whitney U test.

## Data Availability

The data presented in this study are available on request from the corresponding author.
